# Prognostic value of TAPSE in patients with septic cardiomyopathy: a retrospective observational cohort study

**DOI:** 10.3389/fmed.2025.1632964

**Published:** 2025-07-14

**Authors:** Haiyun Liu, Huimin He, Zhongbao Lin, Xiankun Lin, Linqian Jiang, Long Huang, Xiuling Shang, Xincai Wang

**Affiliations:** ^1^Department of Emergency Medicine, Fuzhou University Affiliated Provincial Hospital, Fujian Provincial Hospital, Shengli Clinical Medical College of Fujian Medical University, Fuzhou, China; ^2^Department of Obstetrics and Gynecology, Jin'an Hospital, Fuzhou, China; ^3^Department of Critical Care Medicine, Fuzhou University Affiliated Provincial Hospital, Fujian Provincial Hospital, Shengli Clinical Medical College of Fujian Medical University, Fujian Provincial Center for Critical Care Medicine, Fujian Provincial Key Laboratory of Critical Care Medicine, Fuzhou, China

**Keywords:** tricuspid annular plane systolic excursion (TAPSE), sepsis, septic cardiomyopathy, right heart function, 28-day mortality

## Abstract

**Background:**

Septic cardiomyopathy is an acute myocardial injury caused by sepsis with high mortality. Tricuspid annular plane systolic excursion (TAPSE) is an important parameter for evaluating right ventricular systolic function, but its relationship with prognosis in septic cardiomyopathy has not been clearly elucidated.

**Methods:**

This retrospective cohort study included 93 patients diagnosed with septic cardiomyopathy admitted to the intensive care unit (ICU). All patients underwent TAPSE measurements within 24 h of admission and were divided into abnormal (TAPSE < 1.6 cm, *n* = 33) and normal (TAPSE ≥ 1.6 cm, *n* = 60) groups. The primary outcome was 28-day all-cause mortality. Kaplan–Meier survival analysis was used to compare survival rates between the two groups, and Cox proportional hazards regression models were used to assess the independent association between TAPSE and 28-day mortality. Receiver operating characteristic (ROC) curve analysis was used to evaluate the predictive value of TAPSE for prognosis in septic cardiomyopathy.

**Results:**

Among the 93 patients, 25 (26.8%) died within 28 days after ICU admission. The mean TAPSE value in non-survivors was significantly lower than in survivors (1.24 ± 0.21 vs. 1.72 ± 0.20 cm, *p* < 0.001). Multivariate Cox regression analysis showed that, after adjusting for age, sex, lactate level, APACHE II score, left ventricular ejection fraction, and troponin I, TAPSE was independently associated with 28-day mortality. Dose–response relationship analysis indicated a negative linear relationship between TAPSE values and 28-day mortality, with each 0.1 cm decrease in TAPSE associated with a 40% increase in mortality risk (HR = 0.6, 95% CI: 0.48–0.74, *p* < 0.001). ROC curve analysis showed that TAPSE had an AUC of 0.881 (95% CI: 0.800–0.963) for predicting 28-day mortality, with the cut-off determined by Youden’s index of 1.55 cm, corresponding to a sensitivity of 80.0% and specificity of 80.9%.

**Conclusion:**

TAPSE is independently associated with 28-day mortality in patients with septic cardiomyopathy and can serve as a valuable indicator for assessing short-term prognosis in these patients. Incorporating TAPSE assessment into routine examinations for septic cardiomyopathy patients may assist in identifying high-risk patients early for clinical monitoring.

## Introduction

Sepsis is a life-threatening organ dysfunction caused by a dysregulated host response to infection, which can affect multiple organs, leading to organ dysfunction or failure (1, 2). Globally, approximately 27–30 million people develop sepsis annually, with mortality rates ranging from 10 to 52% (3). Septic cardiomyopathy (SCM) is a common complication of sepsis, characterized by acute myocardial dysfunction in the absence of primary heart disease (4), reported in over 50% of sepsis patients (5).

Despite significant advances in intensive care treatment, SCM remains an important cause of morbidity and mortality in critically ill patients (6). Traditionally, the diagnosis and prognostic assessment of SCM have primarily focused on left heart function, with relatively less attention paid to right heart function. However, the importance of right heart function in critically ill patients has gained recognition in recent years, with studies showing that nearly half of patients with sepsis and septic shock have right ventricular dysfunction, which is associated with more than a threefold increase in 28-day mortality (7).

Tricuspid annular plane systolic excursion (TAPSE) is a simple, easily obtained, and reproducible echocardiographic parameter that reliably reflects right ventricular systolic function (8). In various cardiovascular diseases, TAPSE has been proven to be a valuable prognostic marker (9–11). However, the role of TAPSE in predicting prognosis in patients with septic cardiomyopathy has not been thoroughly studied.

This study aims to investigate whether right ventricular dysfunction, as assessed by TAPSE, can provide additive prognostic value in patients with septic cardiomyopathy defined by conventional left ventricular-based diagnostic criteria. Specifically, we sought to determine the association between TAPSE and 28-day mortality in this patient population and to evaluate the clinical utility of TAPSE as a complementary prognostic assessment tool beyond traditional left ventricular parameters, thereby providing a basis for enhanced risk stratification and treatment decisions in patients with septic cardiomyopathy.

## Methods

### Study design and population

This single-center retrospective cohort study was approved by the Ethics Committee of Fujian Provincial Hospital (approval number: K2018-09-056), with the requirement for informed consent waived due to the retrospective nature of the study. The study included adult patients (≥18 years) admitted to the intensive care unit (ICU) of Fujian Provincial Hospital between January 2018 and December 2022 who were diagnosed with septic cardiomyopathy.

The diagnostic criteria for septic cardiomyopathy were: (1) confirmed infection and an acute increase in Sequential Organ Failure Assessment (SOFA) score ≥2 points from baseline (baseline SOFA score was assumed to be zero for patients without known preexisting organ dysfunction) (1); (2) cardiac ultrasound showing reduced ventricular systolic function (left ventricular ejection fraction [LVEF-S] < 45%) and/or left ventricular dilatation (left ventricular internal diameter > 55 mm); (3) elevated cardiac markers (B-type natriuretic peptide [BNP], troponin I [cTnI]). Exclusion criteria were: (1) age < 18 years; (2) active diseases directly related to myocardial dysfunction, such as acute myocardial infarction, unstable arrhythmias, and post-cardiopulmonary resuscitation status; (3) patients with significant underlying heart disease, such as congenital heart disease and valvular heart disease; (4) patients who underwent cardiac surgery within 2 months; (5) pregnant and lactating patients; (6) inability to complete echocardiography in a timely manner; (7) unwillingness to participate in the study or unexpected discharge.

### Data collection

The following information was collected from the electronic medical record system: (1) demographic characteristics: age, sex; (2) underlying diseases: diabetes mellitus, hypertension, chronic obstructive pulmonary disease (COPD), chronic kidney disease (CKD), immunosuppressive status; (3) clinical severity scores: Acute Physiology and Chronic Health Evaluation II (APACHE II), SOFA score; (4) laboratory indicators: N-terminal pro-brain natriuretic peptide (NT-proBNP), troponin I (cTnI), lactate, procalcitonin (PCT), lactate dehydrogenase (LDH), creatine kinase (CK), anion gap (AG); (5) clinical treatment measures: mechanical ventilation, use of vasoactive drugs; (6) clinical outcomes: ICU length of stay, 28-day mortality. All patients in the final cohort had complete echocardiographic and clinical data for the analyzed variables, as these were routinely collected as part of standard ICU care.

### Echocardiographic examination

All patients underwent echocardiographic examination within 24 h after ICU admission by trained ultrasound specialists using an EDGE-type color Doppler ultrasound machine (Sono Sound). The examination was performed with the patient in the left lateral decubitus position to obtain the standard apical four-chamber view. Measured parameters included: left ventricular internal diameter (LVID), left ventricular ejection fraction-Simpson’s method (LVEF-S), and TAPSE.

TAPSE was obtained as follows: in the apical four-chamber view, the ultrasound equipment was adjusted to M-mode, with the sampling line placed at the lateral tricuspid annulus, simultaneously displaying real-time B-mode and M-mode images. The TAPSE value was determined by measuring the maximum longitudinal displacement of the tricuspid annulus from end-diastole to end-systole in a single cardiac cycle. Normal TAPSE value was defined as ≥1.6 cm, with <1.6 cm considered abnormal (7).

### Statistical analysis

Continuous variables were expressed as mean ± standard deviation (SD) or median (interquartile range [IQR]) according to distribution characteristics, and categorical variables were expressed as frequency (percentage). For comparison of continuous variables between groups, Student’s *t*-test or Mann–Whitney U test was used according to the normality of data distribution, and chi-square test or Fisher’s exact test was used for categorical variables.

Kaplan–Meier method was used to plot survival curves, and Log-rank test was used to compare survival rates between normal and abnormal TAPSE groups. Univariate and multivariate Cox proportional hazards regression models were used to analyze the association between TAPSE and 28-day mortality, with results expressed as hazard ratio (HR) and 95% confidence interval (CI). Potential confounding factors included in the multivariate model were age, sex, lactate level, APACHE II score, LVEF-S, and cTnI (6 covariates in total). The final multivariate model included these 6 covariates with 25 observed events (deaths), resulting in an events-per-variable (EPV) ratio of 4.17. In addition, we used a four-node restricted cubic spline function (nodes at the 5th, 35th, 65th, and 95th percentiles of TAPSE distribution) to assess the dose–response relationship between TAPSE and mortality risk.

Receiver operating characteristic (ROC) curve analysis was used to assess the predictive value of TAPSE for 28-day mortality, calculating the area under the curve (AUC), sensitivity, and specificity. DeLong’s method was used to compare the AUCs of TAPSE, APACHE II score, and LVEF-S as prognostic indicators.

Statistical analysis was performed using R software (version 3.3.2, http://www.R-project.org) and Free Statistics software (version 1.9.2). *p* < 0.05 was considered statistically significant.

Sample size calculation was based on previously reported 28-day mortality in septic cardiomyopathy patients (approximately 30%) and TAPSE abnormality rate (approximately 35%) (6). Using *α* = 0.05 (two-sided) and 80% power, it was estimated that at least 90 patients needed to be included to detect a clinically meaningful difference with a hazard ratio of 2.5.

## Results

### Study population characteristics

**Figure 1:**
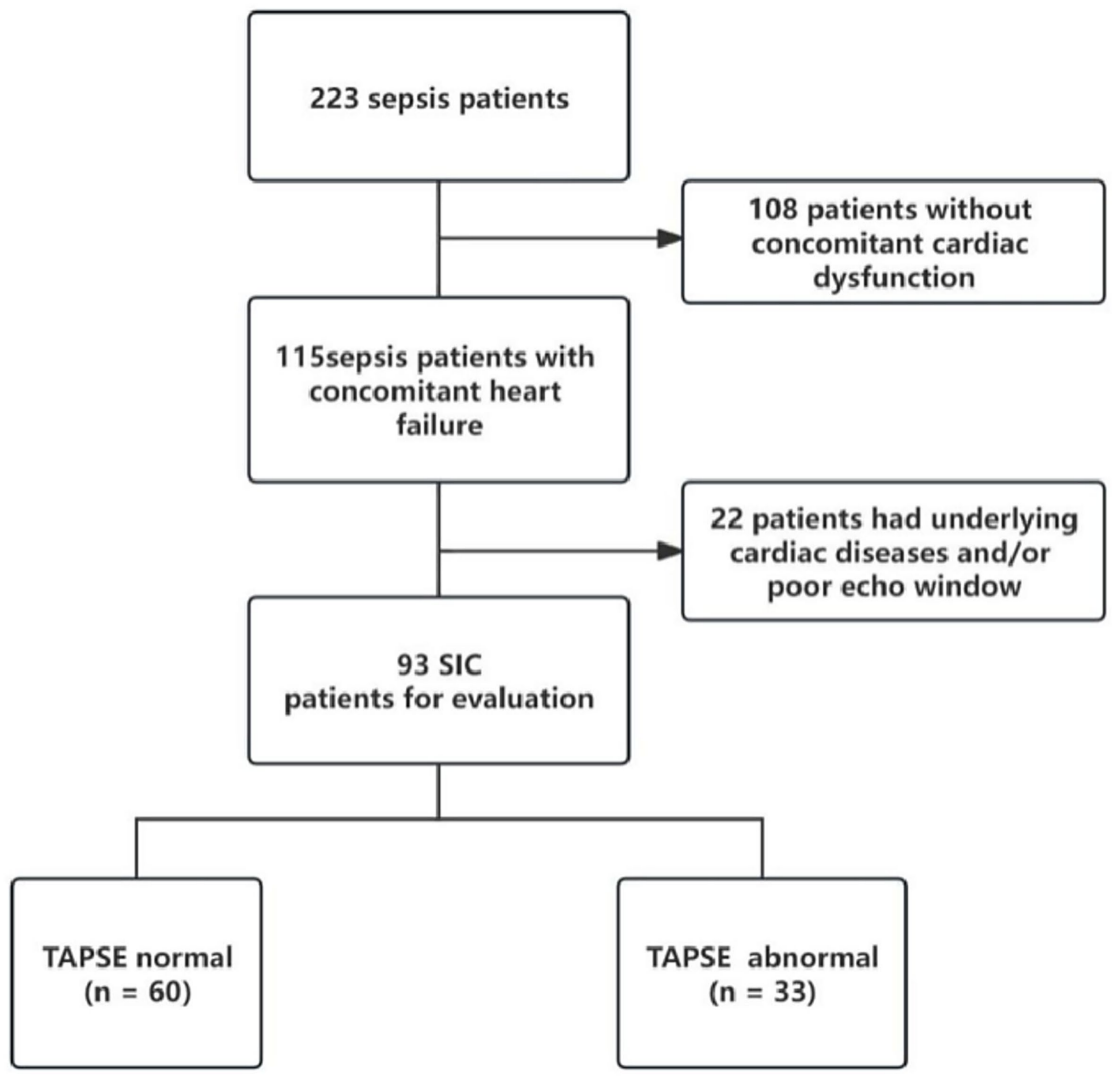
Patient screening flow diagram.

During the study period, there were 115 sepsis patients with heart dysfunction, of whom 93 met the inclusion criteria for septic cardiomyopathy and completed echocardiographic examination (Figure 1). The mean age of patients was 64.8 ± 14.7 years, and 63.4% were male. According to TAPSE values, 33 patients (35.5%) were classified as the abnormal TAPSE group (<1.6 cm) and 60 patients (64.5%) as the normal TAPSE group (≥1.6 cm).

Table 1 shows the baseline characteristics of the study population. Compared with the normal TAPSE group, patients in the abnormal TAPSE group were older (71.1 ± 9.1 vs. 61.4 ± 16.1 years, *p* = 0.002), had lower LVEF-S (44.0 ± 6.7% vs. 49.0 ± 6.2%, *p* < 0.001), higher APACHE II scores (28.8 ± 6.8 vs. 23.9 ± 5.5, *p* < 0.001), and higher lactate levels (4.0 ± 2.1 vs. 3.2 ± 1.7 mmol/L, *p* = 0.047). There were no significant differences between the two groups in terms of sex, underlying diseases, NT-proBNP, cTnI, SOFA score, PCT, mechanical ventilation, and use of vasoactive drugs.

**Table 1:** Baseline characteristics of study participants stratified by TAPSE status.

Characteristics	Total (*n* = 93)	Level of TAPSE		*p* value

		Abnormal (*n* = 33)	Normal (*n* = 60)	
Demographics				
Age, years*	64.8 ± 14.7	71.1 ± 9.1	61.4 ± 16.1	0.002
Male sex, *n* (%)	59 (63.4)	20 (60.6)	39 (65.0)	0.674
Comorbidities, n (%)				
Diabetes mellitus	28 (30.1)	14 (42.4)	14 (23.3)	0.055
Hypertension	43 (46.2)	15 (45.5)	28 (46.7)	0.911
COPD	5 (5.4)	2 (6.1)	3 (5.0)	>0.999
CKD	8 (8.6)	4 (12.1)	4 (6.7)	0.448
Immunosuppression	1 (1.1)	0 (0)	1 (1.7)	>0.999
Cardiac parameters				
LVID, cm*	54.1 ± 3.5	55.0 ± 3.6	53.7 ± 3.3	0.076
TAPSE, cm*	1.6 ± 0.3	1.2 ± 0.2	1.7 ± 0.2	<0.001
LVEF-S, %*	47.2 ± 6.8	44.0 ± 6.7	49.0 ± 6.2	<0.001
Laboratory values				
NT-proBNP, pg./mL†	3500.0 (1842.0, 6203.0)	4186.0 (2150.0, 6771.0)	3456.0 (1581.5, 5386.2)	0.243
cTnI, ng/mL*	0.5 ± 1.0	0.8 ± 1.4	0.4 ± 0.7	0.073
Lactate, mmol/L*	3.5 ± 1.9	4.0 ± 2.1	3.2 ± 1.7	0.047
PCT, ng/mL*	36.7 ± 35.5	41.4 ± 35.0	34.1 ± 35.8	0.347
LDH, IU/L†	334.0 (223.0, 673.0)	344.0 (256.0, 439.0)	312.0 (206.0, 674.2)	0.315
AG, mmol/L*	18.9 ± 6.4	19.5 ± 7.9	18.5 ± 5.4	0.463
CK, IU/L†	178.0 (66.0, 465.0)	150.0 (69.0, 949.0)	178.0 (65.2, 307.2)	0.505
Disease severity				
APACHE II score*	25.6 ± 6.4	28.8 ± 6.8	23.9 ± 5.5	<0.001
SOFA score*	6.8 ± 1.4	6.5 ± 1.4	6.9 ± 1.4	0.171
Clinical management				
Mechanical ventilation, *n* (%)	14 (15.1)	7 (21.2)	7 (11.7)	0.238
Vasopressor requirement, *n* (%)	82 (88.2)	31 (93.9)	51 (85.0)	0.317
ICU length of stay, days*	7.0 (5.0,12.0)	7.0 (4.0, 12.0)	7.0 (5.0, 11.2)	0.234

*Data are presented as mean ± SD. †Data are presented as median (IQR). TAPSE, tricuspid annular plane systolic excursion; COPD, chronic obstructive pulmonary disease; CKD, chronic kidney disease; LVID, left ventricular internal diameter; LVEF-S, left ventricular ejection fraction-Simpson; NT-proBNP, N-terminal pro-brain natriuretic peptide; cTnI, cardiac troponin I; PCT, procalcitonin; LDH, lactate dehydrogenase; AG, anion gap; CK, creatine kinase; APACHE, Acute Physiology and Chronic Health Evaluation; SOFA, Sequential Organ Failure Assessment; ICU, intensive care unit.

### Risk factors and survival analysis

Univariate Cox regression analysis showed that age, TAPSE, LVEF-S, cTnI, APACHE II score, and lactate level were significant predictors of 28-day mortality (Table 2). Among the 93 patients, 25 (26.8%) died within 28 days, and these six covariates were subsequently included in the multivariate analysis. In multivariate Cox regression analysis, TAPSE was analyzed as both a continuous variable and a categorical variable (normal vs. abnormal). As shown in Table 3, after adjusting for potential confounding factors, TAPSE remained independently associated with 28-day mortality. In the final model (Model 4), each 0.1 cm decrease in TAPSE was associated with a 40% increase in mortality risk (adjusted HR = 0.6, 95% CI: 0.48–0.74, *p* < 0.001); compared with the normal TAPSE group, patients in the abnormal TAPSE group had a 7.55-fold increase in mortality risk (adjusted HR = 8.55, 95% CI: 2.91–25.15, *p* < 0.001).

**Table 2:** Univariate analysis of risk factors associated with 28-day mortality.

Variable	HR (95% CI)	*p* value
Demographics		
Gender, male	0.73 (0.33, 1.60)	0.428
Age, years	1.05 (1.01, 1.09)	0.008
Comorbidities		
Diabetes	1.65 (0.74, 3.67)	0.223
Immunosuppression	NA*	0.998
Hypertension	1.53 (0.69, 3.37)	0.293
COPD	NA*	0.997
CKD	1.49 (0.45, 4.99)	0.516
Cardiac parameters		
LVID, cm	1.07 (0.95, 1.21)	0.286
TAPSE, cm	0.01 (0.00, 0.04)	<0.001
LVEF-S, %	0.83 (0.79, 0.88)	<0.001
Laboratory values		
NT-proBNP, pg./mL†	1.00 (1.00, 1.0001)	0.130
cTnI, ng/mL	1.58 (1.25, 1.99)	<0.001
APACHE II score	1.24 (1.16, 1.34)	<0.001
SOFA score	0.89 (0.67, 1.19)	0.434
Lactate, mmol/L	1.22 (1.02, 1.46)	0.026
PCT, ng/mL	1.008 (0.998, 1.019)	0.110
LDH, IU/L	1.000 (1.000, 1.000)	0.749
AG, mmol/L	1.04 (0.99, 1.11)	0.127
CK, IU/L	1.000 (1.000, 1.000)	0.747

* NA indicates hazard ratio could not be calculated due to zero events in one group. † HR presented to 4 decimal places due to small effect size. COPD, chronic obstructive pulmonary disease; CKD, chronic kidney disease; LVID, left ventricular internal diameter; TAPSE, tricuspid annular plane systolic excursion; LVEF-S, left ventricular ejection fraction-Simpson; NT-proBNP, N-terminal pro-brain natriuretic peptide; cTnI, cardiac troponin I; PCT, procalcitonin; LDH, lactate dehydrogenase; AG, anion gap; CK, creatine kinase; APACHE, Acute Physiology and Chronic Health Evaluation; SOFA, Sequential Organ Failure Assessment.

**Table 3:** Multivariate Cox regression analysis for TAPSE on 28-day mortality.

Models	TAPSE as continuous* (*N* = 93)		TAPSE as categorical	
	HR (95% CI)	*p* value	Abnormal (*n* = 33) HR (95% CI)	Normal (*n* = 60)
Model 1	0.64 (0.56–0.72)	<0.001	11.61 (4.33–31.12)	1 (Reference)
Model 2	0.65 (0.56–0.75)	<0.001	9.70 (3.61–26.12)	1 (Reference)
Model 3	0.69 (0.59–0.80)	<0.001	7.33 (2.57–20.89)	1 (Reference)
Model 4	0.60 (0.48–0.74)	<0.001	8.55 (2.91–25.15)	1 (Reference)

*TAPSE was analyzed per 0.1 cm decrease as continuous variable. Model 1: Crude model. Model 2: Adjusted for age. Model 3: Adjusted for Model 2 + Lactate + APACHE II score. Model 4: Adjusted for Model 3 + LVEF-S + cTnI. TAPSE, tricuspid annular plane systolic excursion; APACHE, Acute Physiology and Chronic Health Evaluation; LVEF-S, left ventricular ejection fraction-Simpson; cTnI, cardiac troponin I.

**Figure 2:**
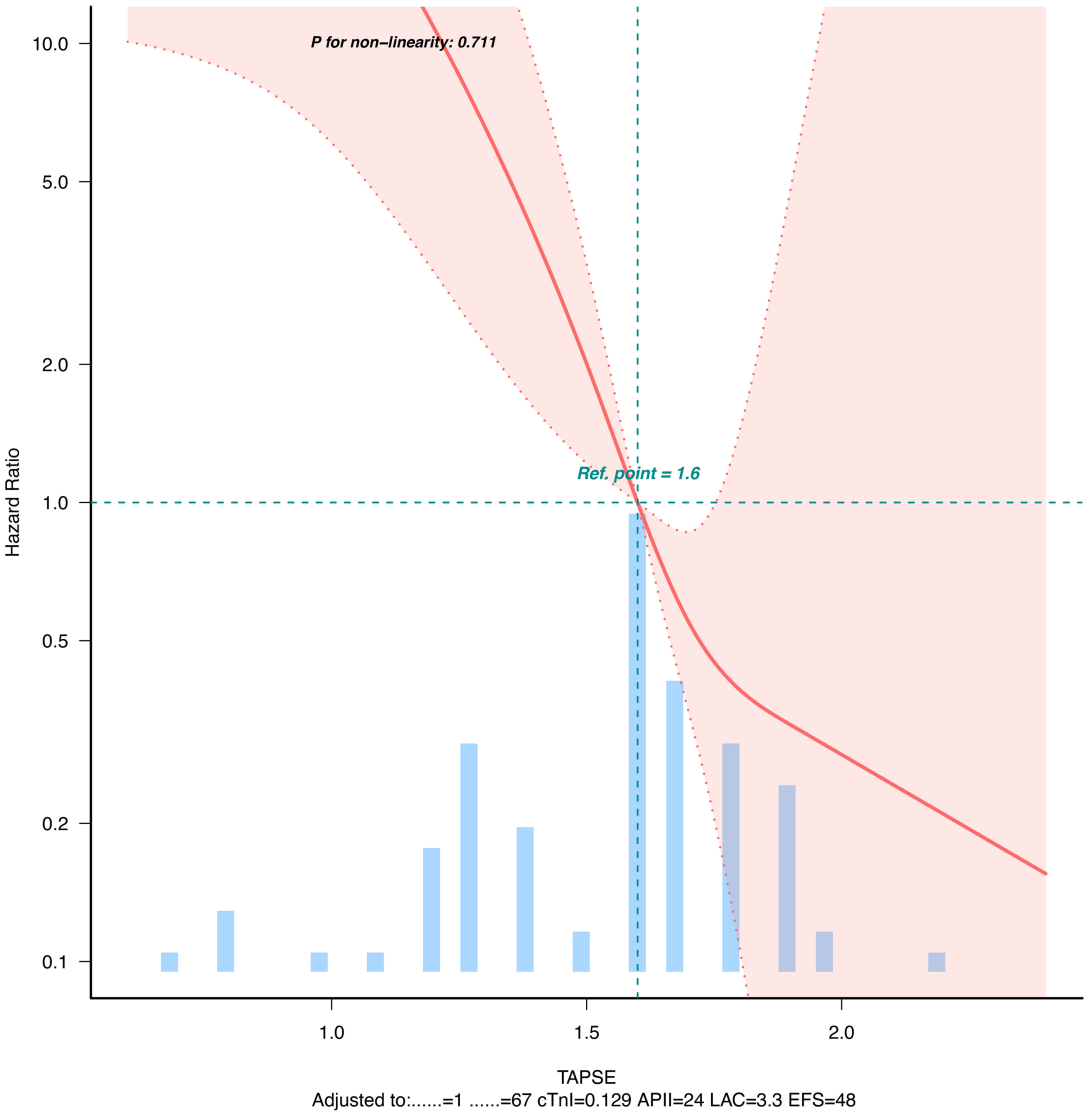
Dose–response relationship curve between TAPSE and 28-day mortality.

Dose–response relationship analysis showed a negative linear relationship between TAPSE and 28-day mortality risk after adjusting for potential confounding factors (Figure 2).

### Association between TAPSE and 28-day mortality

**Figure 3:**
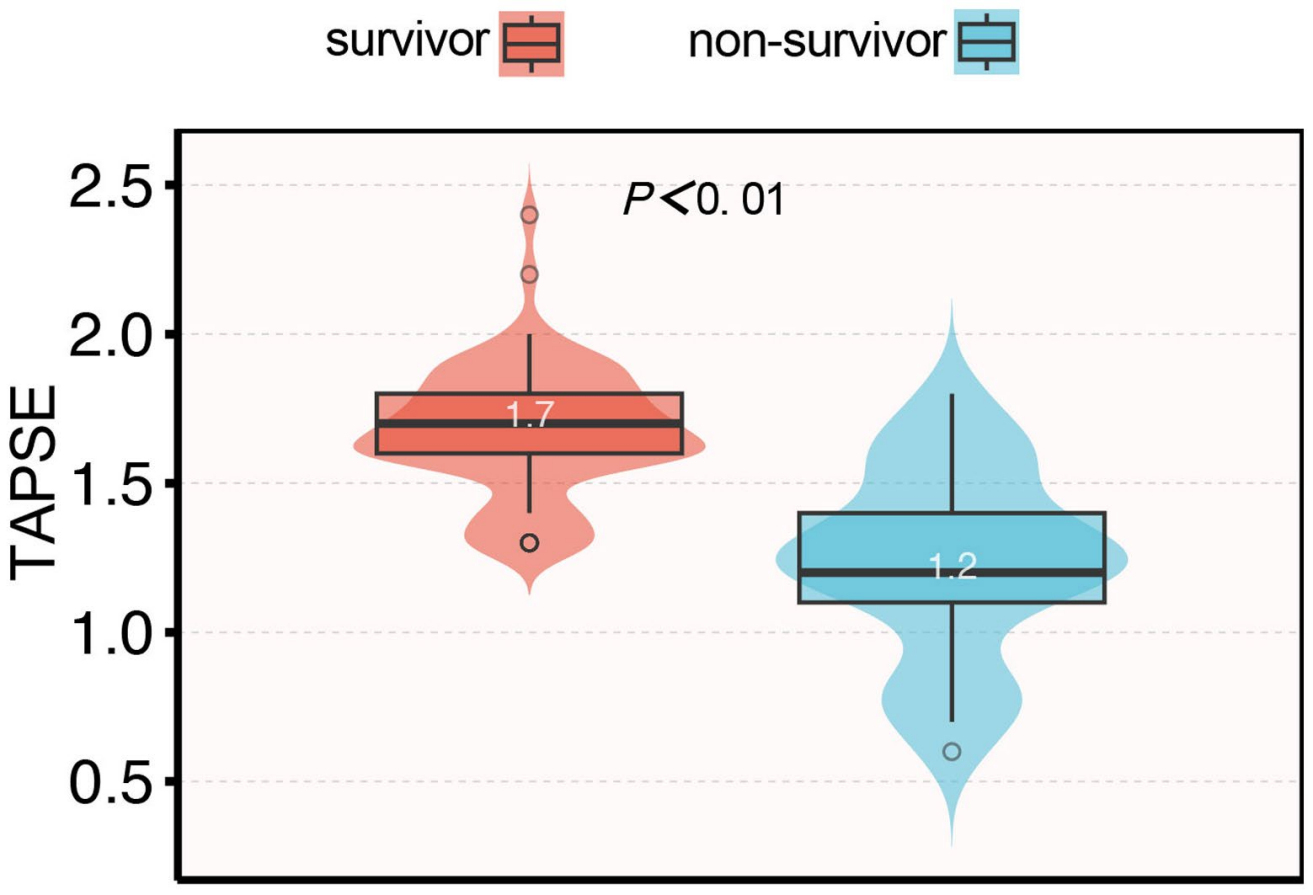
Violin plot of TAPSE values in survivor and non-survivor groups.

**Figure 4:**
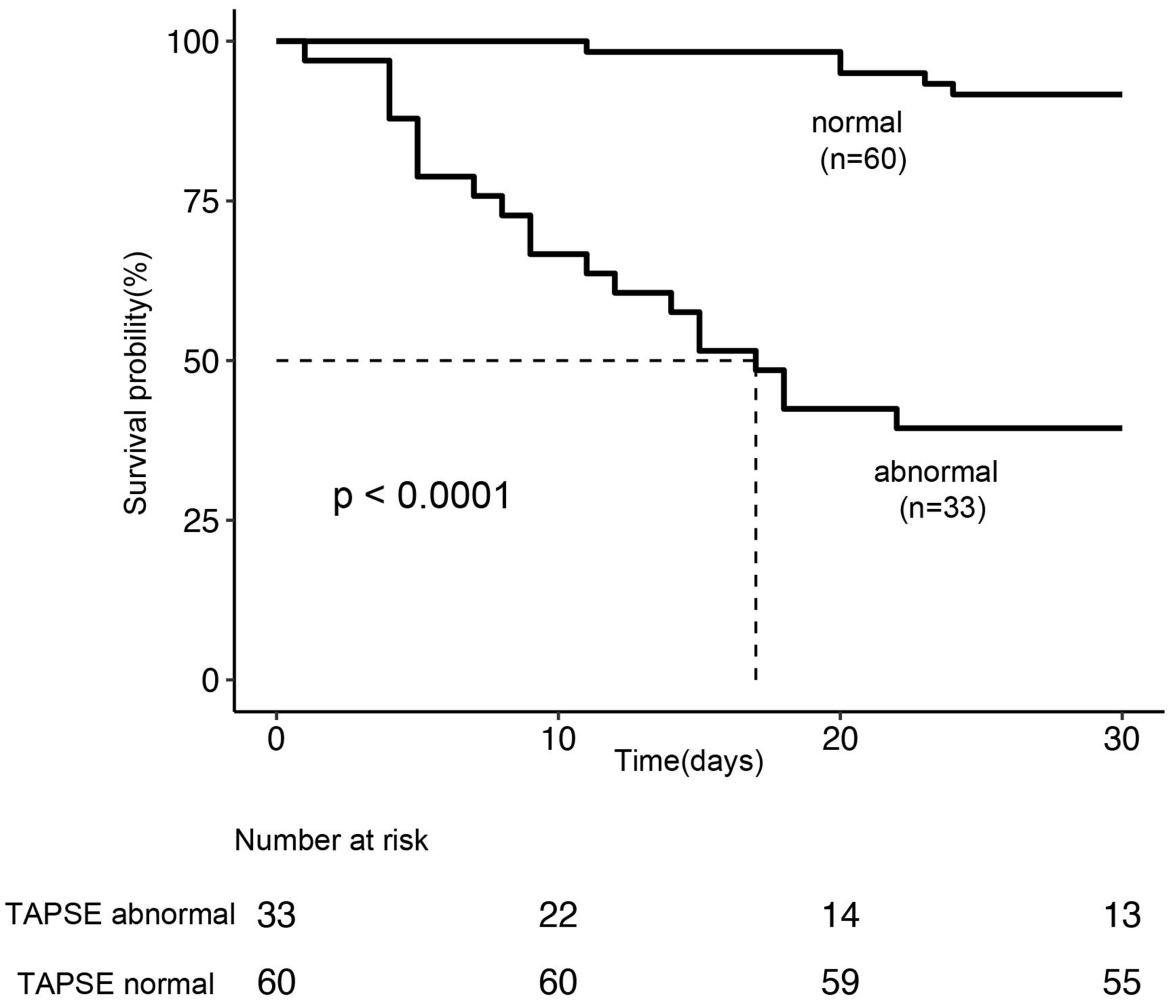
Kaplan–Meier survival curve analysis based on TAPSE grouping (normal vs. abnormal).

Among the 93 patients with septic cardiomyopathy, 25 (26.8%) died within 28 days after ICU admission. The mean TAPSE value in non-survivors was significantly lower than in survivors (1.24 ± 0.21 vs. 1.72 ± 0.20 cm, *p* < 0.001; Figure 3). Kaplan–Meier survival analysis showed that the 28-day survival rate in the abnormal TAPSE group was significantly lower than in the normal TAPSE group (Log-rank test, *p* < 0.001; Figure 4).

**Figure 5:**
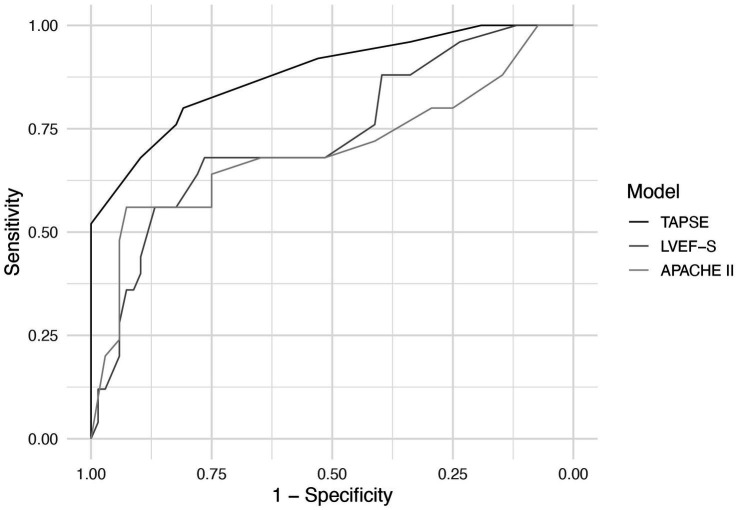
ROC curves of TAPSE, LVEF-S, and APACHE II for predicting 28-day mortality in septic cardiomyopathy.

Receiver operating characteristic (ROC) curve analysis showed that TAPSE had the highest discriminatory ability for predicting 28-day mortality, with an AUC of 0.881 (95% CI: 0.800–0.963), followed by LVEF-S (AUC = 0.734, 95% CI: 0.623–0.845) and APACHE II (AUC = 0.700, 95% CI: 0.570–0.829). The AUC for TAPSE was significantly higher than that of APACHE II (*p* = 0.043). No significant differences were found between LVEF-S and APACHE II (*p* = 0.541), or between TAPSE and LVEF-S (*p* = 0.064), although the latter showed a trend toward significance(Figure 5). The optimal cut-off value of TAPSE, determined by Youden’s index, was 1.55 cm, corresponding to a sensitivity of 80.0% and a specificity of 80.9%.

## Discussion

This study investigated the association between TAPSE and 28-day mortality in patients with septic cardiomyopathy, and the results showed that patients with septic cardiomyopathy with low TAPSE values had a significantly increased risk of 28-day mortality. This association remained after adjusting for potential confounding factors, indicating that TAPSE is an independent prognostic indicator. We found a negative linear relationship between TAPSE and mortality risk, and the predictive value of TAPSE for mortality in septic cardiomyopathy patients was high. These results emphasize the importance of assessing right heart function in patients with septic cardiomyopathy and suggest that TAPSE may be a valuable tool for prognostic assessment in this population.

Septic cardiomyopathy is a complex pathophysiological process that can affect the left ventricle, right ventricle, or both (12). Traditionally, research and definitions of SCM have mainly focused on left ventricular dysfunction, with relatively less attention paid to right heart function. However, the right ventricle plays an important role in maintaining cardiac pump function and systemic circulation, and when the right ventricle is impaired, it can lead to reduced venous return, affecting left heart filling and systemic perfusion (13). In this study, we found that patients with abnormal TAPSE (<1.6 cm) were older, had higher APACHE II scores, and higher lactate levels compared to patients with normal TAPSE, suggesting that right heart dysfunction may be associated with disease severity and poor tissue perfusion. This is consistent with previous research findings, such as Lanspa et al.’s (7) report that right ventricular dysfunction in sepsis patients is associated with microcirculatory perfusion abnormalities and organ dysfunction.

In septic cardiomyopathy, the development of right heart dysfunction involves multiple mechanisms. Inflammatory mediators such as tumor necrosis factor-*α* (TNF-α) and interleukin-1β (IL-1β) (14)can directly inhibit myocardial contractility. Sepsis-associated acute lung injury and hypoxemia can cause pulmonary vasoconstriction, increasing right ventricular afterload (15). Mechanical ventilation, especially high positive pressure ventilation strategies, can increase intrathoracic pressure, reduce venous return, and simultaneously increase pulmonary vascular resistance, further impairing right heart function (16). In addition, vasoactive drugs such as norepinephrine may increase pulmonary vascular resistance, while dobutamine may improve right heart contractility, and drug selection also affects right heart function (17–19). Notably, the anatomical and physiological characteristics of the right ventricle make it particularly sensitive to volume and pressure changes in sepsis. Compared with the left ventricle, the right ventricle has a thinner wall, higher compliance, stronger adaptability to increased preload, but poorer tolerance to increased afterload (20). TAPSE, as an indicator reflecting right ventricular longitudinal contractile function, can sensitively capture these changes, which may explain its superiority in predicting prognosis.

Our study found that patients in the abnormal TAPSE group had significantly higher lactate levels than those in the normal TAPSE group, suggesting that right heart dysfunction may be associated with poor tissue perfusion and oxygen supply–demand imbalance. Right ventricular dysfunction can lead to pulmonary circulation congestion, reduced systemic venous return, and subsequently affect cardiac output and tissue perfusion (21). Lactate is a marker of poor tissue perfusion and anaerobic metabolism, and elevated levels often indicate poor prognosis (22). Notably, right heart dysfunction may affect lactate clearance through hepatic congestion, thereby exacerbating hyperlactatemia (23, 24). Therefore, hyperlactatemia in septic cardiomyopathy may reflect both increased lactate production due to poor tissue perfusion and decreased lactate clearance due to reduced hepatic clearance capacity, forming a vicious cycle. This emphasizes the importance of early identification and intervention of right heart dysfunction.

Although this study focused on the role of TAPSE in prognostic assessment of septic cardiomyopathy, echocardiography provides multiple parameters for evaluating right heart function. The TAPSE/systolic pulmonary artery pressure (PASP) ratio, as an indicator of right ventricular-pulmonary arterial coupling, has received increasing attention in recent years (25). Schmeisser et al. (9) validated through pressure-volume loops that TAPSE/PASP reliably reflects the right ventricular-pulmonary arterial coupling state, especially in heart failure patients with pulmonary hypertension (9). In addition, right ventricular fractional area change (RVFAC), tricuspid annular systolic velocity (S′), and right ventricular free wall longitudinal strain (RVLS) are also effective indicators for evaluating right ventricular systolic function (26–28). Compared with these indicators, the advantage of TAPSE lies in its simplicity of measurement, good reproducibility, and no need for special techniques or software, making it particularly suitable for use in emergency situations and ICU environments. The results of this study suggest that TAPSE can be an effective tool for early risk stratification in patients with septic cardiomyopathy. Patients with TAPSE < 1.6 cm may need more aggressive monitoring and therapeutic interventions, including optimizing volume status, adjusting ventilator parameters to reduce right heart afterload, and selecting appropriate vasoactive drugs.

Several limitations of this study need to be considered. First, this is a single-center retrospective study with inherent selection bias and confounding factors that cannot be fully controlled. Additionally, the relatively small cohort precluded meaningful subgroup analyses based on infection source, severity of illness, or specific therapeutic interventions. Furthermore, the limited sample size may increase the risk of overfitting in our multivariate regression models, potentially affecting the generalizability of our findings. Larger multicenter cohort studies are needed to validate our results and enable more robust subgroup analyses.

Second, we only performed TAPSE measurement once within 24 h after ICU admission, lacking continuous monitoring data. Dynamic changes in TAPSE may provide more prognostic information, especially its value in assessing treatment response. Harmankaya et al. showed that in patients with septic shock, dynamic changes in right heart function parameters are closely related to prognosis, and static single measurements may not capture complete information (29).

Third, our multivariate model did not incorporate critical therapeutic factors known to significantly influence right ventricular function, such as types of vasoactive medications (e.g., norepinephrine and dobutamine), fluid management strategies, and ventilator settings. Previous studies have demonstrated that these interventions can substantially alter right ventricular loading conditions and contractility, thereby affecting TAPSE measurements. For instance, norepinephrine may increase right ventricular afterload, while dobutamine can enhance contractility; fluid management and ventilatory adjustments significantly influence preload and afterload. Consequently, the omission of these therapeutic factors might have introduced confounding into our results, potentially affecting the robustness of our conclusions. Future studies should systematically document and integrate these treatment variables into multivariate models to comprehensively evaluate their specific impact on the prognostic value of TAPSE.

Additionally, this study did not evaluate other established right heart function parameters, such as TAPSE/PASP ratio, RVFAC, S′, and RVLS, limiting our ability to determine the optimal echocardiographic approach for right ventricular assessment in septic cardiomyopathy. We strongly recommend that future prospective investigations incorporate comprehensive right ventricular evaluation protocols to compare the individual and combined prognostic value of these parameters in septic cardiomyopathy patients.

Moreover, it is important to acknowledge that our study focused on patients diagnosed with septic cardiomyopathy based on left ventricular dysfunction criteria, which represents a significant limitation. Patients with isolated right ventricular dysfunction who did not meet our left ventricular-based diagnostic criteria were excluded, potentially underrepresenting sepsis-related right heart dysfunction. However, our approach of evaluating right ventricular function (TAPSE) in patients defined by conventional left ventricular-based SCM criteria represents a transitional step toward advocating for more comprehensive biventricular diagnostic frameworks in future SCM definitions. By demonstrating the independent prognostic value of right heart assessment in this population, our findings support the clinical rationale for evolving current diagnostic paradigms. Future studies should therefore incorporate comprehensive biventricular assessment criteria to capture the full spectrum of septic cardiomyopathy.

Finally, although we found TAPSE to be associated with prognosis, we cannot determine whether this association is causal or merely associative. It is important to acknowledge that low TAPSE values may reflect global severity of illness rather than isolated right ventricular dysfunction. In this context, TAPSE may serve as a marker of overall disease severity rather than a direct cause of death. Only targeted intervention studies can determine whether improving right heart function can improve prognosis.

## Conclusion

This study demonstrates that TAPSE is independently associated with 28-day mortality in patients with septic cardiomyopathy and has good predictive value. TAPSE, as a simple and reliable indicator of right heart function assessment, can be used for early risk stratification and prognostic assessment in patients with septic cardiomyopathy. We recommend incorporating TAPSE assessment into routine examinations for patients with septic cardiomyopathy to identify high-risk patients early and optimize treatment strategies. Future prospective multicenter studies are needed to further validate the predictive value of TAPSE and explore whether TAPSE-guided risk stratification and targeted interventions are associated with improved outcomes in patients with septic cardiomyopathy.

## Data Availability

The raw data supporting the conclusions of this article will be made available by the authors, without undue reservation.

## Glossary

AG: Anion gap
APACHE II: Acute Physiology and Chronic Health Evaluation II
AUC: Area under the curve
BNP: B-type natriuretic peptide
CI: Confidence interval
CK: Creatine kinase
CKD: Chronic kidney disease
COPD: Chronic obstructive pulmonary disease
cTnI: Cardiac troponin I
HR: Hazard ratio
ICU: Intensive care unit
IQR: Interquartile range
LDH: Lactate dehydrogenase
LVEF-S: Left ventricular ejection fraction-Simpson’s method
LVID: Left ventricular internal diameter
NT-proBNP: N-terminal pro-brain natriuretic peptide
PASP: Pulmonary artery systolic pressure
PCT: Procalcitonin
ROC: Receiver operating characteristic
RVFAC: Right ventricular fractional area change
RVLS: Right ventricular free wall longitudinal strain
SCM: Septic cardiomyopathy
SD: Standard deviation
SOFA: Sequential Organ Failure Assessment
TAPSE: Tricuspid annular plane systolic excursion
TNF-*α*: Tumor necrosis factor-alpha
